# Responding to Older Adult Maltreatment: Interdisciplinary Geriatric Care Provider Experiences and Training Needs

**DOI:** 10.1177/08445621261443833

**Published:** 2026-04-20

**Authors:** Joshua Wyman, Cassandre Dion Larivière, Tala Tayem, Lindsay Malloy

**Affiliations:** 1 Department of Psychology, 113609 King's University College at Western University, London, Canada; 2 Faculty of Social Sciences and Humanities, 418638 Ontario Tech University, Oshawa, Canada; 3 Department of Psychology, 418638 University of Waterloo, Waterloo, Canada; 4 Faculty of Social Sciences and Humanities, Ontario Tech University, Oshawa, Canada

**Keywords:** Older adults, maltreatment, Canada, geriatric care providers, long-term care, professional development

## Abstract

In the current mixed methods study, 37 interdisciplinary geriatric care providers in Ontario, Canada, completed a qualitative interview and a series of quantitative questionnaires. The qualitative interview explored their experiences observing, identifying and reporting older adult maltreatment in their work settings, along with their training background and recommendations for professional development. The quantitative measures assessed their abilities to identify risk factors for older adult maltreatment, and their attitudes and willingness to assess for potential indicators of maltreatment. Inductive thematic coding of the qualitative interviews revealed that these providers frequently observe older adult maltreatment in their workplaces, which is most often perpetrated by family members and geriatric care providers. Several barriers to reporting older adult maltreatment were identified, including fear of consequences, older adult apprehension, insufficient reporting knowledge, training and preparedness, and professional and institutional barriers. Although most providers accurately identified the common risk factors for older adult maltreatment on the quantitative measures, they nevertheless expressed clear individual, institutional and professional barriers to maltreatment reporting during the qualitative interviews. These findings underscore the essential need for a culture change in reporting processes, professional and institutional support, training and psychological safety to ensure that all interdisciplinary geriatric care providers have the confidence and preparation to effectively assist older adults who are at-risk for abuse and neglect.

Interdisciplinary geriatric care providers, including nurses, personal support workers (PSWs), physicians, social workers and other multidisciplinary professionals (e.g., psychologists and occupational therapists), are often witnesses and disclosure recipients of maltreatment involving older adults (Yon et al., 2019). Despite there being mandatory reporting laws in Ontario, Canada, for older adult residents in community, retirement home and long-term care (LTC) settings, interdisciplinary geriatric care providers can be reluctant to disclose observed maltreatment due to insufficient training and time (Reingle Gonzalez et al., 2016; Schmeidel et al., 2012). Failure to report observed maltreatment can expose older adults to serious, escalating, and potentially life-threatening harm (Burnett et al., 2016; Dong et al., 2009). In their review of the scientific literature, Atkinson and Roberto (2024) argued that addressing the barriers to detecting and reporting older adult maltreatment is essential for preventing future victimization.

The current mixed methods study was designed to better comprehend why interdisciplinary geriatric care providers in Ontario, Canada, may be reluctant or unable to effectively report older adult maltreatment, as well as to inform future professional development programs. The first objective of this study was to explore interdisciplinary geriatric care provider experiences observing older adult maltreatment in their workplaces, including their perceptions of the common indicators, risk factors and perpetrators. The second objective involved gathering insight into the older adult-specific and professional barriers to reporting older adult maltreatment. The third objective was to investigate interdisciplinary geriatric care provider preparedness to respond to situations involving older adult maltreatment, along with their recommendations for future professional development. The results from this study can inform interdisciplinary geriatric care provider training, professional and institutional policies, and reporting guidelines for assisting older adults who are most at-risk for being maltreated.

## The Aging Canadian Population

The older adult population, which includes those aged 65 years and older (Government of Canada, 2017), is the fastest growing age demographic in Canada. The percentage of older adults is expected to increase from 18.9% of the population in 2024 to between 21.6% (slow-aging projection) to 31.7% (fast aging projection) of the population by 2073 (Statistics Canada, 2025). The number of people aged 85 years and older in 2021 was more than twice the number recorded in the 2001 census (Hallman et al., 2022), and this age group is expected to triple between 2023 and 2073 (Statistics Canada, 2025). The significant increase in the population share of this age group is attributed to the aging baby-boomer generation, coupled with the gradually lower fertility rates of the subsequent generations (Public Health Agency of Canada, 2020). While many older adults live relatively independently well into their 80s and 90s, there is an increasing number of older adults who require care in health, retirement home, nursing home, and community settings. The Conference Board of Canada (2017) projects that Canada will need to nearly double its LTC capacity by 2035 given the need for an additional 199,000 beds.

## Older Adult Maltreatment

The Government of Canada (2021) estimates that approximately 10% of older adults are maltreated every year in Canada, with 45% of older adults experiencing some form of abuse or neglect at some point after the age of 65. Globally, the estimated prevalence rate of older adult maltreatment is 15.7% according to a meta-analysis of 52 prevalence studies (Yon et al., 2017). Psychological abuse is the most prevalent form of older adult maltreatment, followed by financial exploitation, neglect, physical abuse and sexual abuse (Yon et al., 2017). These prevalence rates are an under-estimate given that the majority of maltreatment cases go unreported. For example, Lachs and Berman (2011) interviewed 4,156 older adults and their proxies living in the state of New York, along with 292 community agencies, to gather estimates regarding the prevalence and incidence of older adult maltreatment. Overall, they found that for every one case of older adult maltreatment that went referred to social service, law enforcement or legal authorities, approximately 23.5 self-reported cases went unreferred. This was especially evident for self-reported cases of neglect (1 out of 57.2 cases) and financial exploitation (1 out of 43.9 cases) that went unreferred (Lachs & Berman, 2011).

Baumann et al. (2024) examined the frequency of reported cases of older adult maltreatment in LTC settings in Ontario, Canada. The Ministry of Long-Term Care conducts mandatory and unannounced inspections of LTC homes to assess that they are being operated in compliance with provincial legislation and regulatory standards (Fixing Long-Term Care Act, 2021). Baumann et al. (2024) reviewed publicly available LTC inspection reports that were published by the Ontario Ministry of Long- Term Care. From 2019 to 2022, 781 cases of older adult maltreatment (or 9% of all inspections) were discovered following the review of inspection reports of 627 LTC homes. Staff-to-resident abuse was evident in 56% of these cases, with 62% of these cases involving unregulated staff compared to 10% of cases involving regulated staff. While Baumann et al. (2024) focused on the prevalence of documented cases of older adult maltreatment, the estimates derived from geriatric care provider self reports of older maltreatment is substantially higher. Yon et al. (2019) reviewed nine prevalence studies of older adult maltreatment in institutional settings, including studies from the United States, Germany, Czech Republic, Ireland and Israel. Across these nine studies, 64.2% of institutional staff reported that they engaged in at least one act of older adult maltreatment within the previous year (Yon et al., 2019). It is worth noting that self-reports of staff behavior have limitations, including differences in definitions of older adult maltreatment, severity of behavior, and reporting biases across the nine prevalence studies.

Irrespective of age, maltreatment can cause physical (e.g., lacerations, bruises, fractures, malnutrition and death), psychological (e.g., trauma, anxiety, despair and low self-worth) and/or financial (e.g., loss of savings) suffering (Collins, 2006; Nguyen et al., 2021). Maltreatment can be particularly harmful to older adults, including increased likelihood of hospitalization (Dong et al., 2013), LTC placement (Lachs & Pillemer, 2015), and mortality risk for older adults who have been maltreated (Burnett et al., 2016; Dong et al., 2009). Older adults face an increased risk of harm due to preexisting medical conditions, functional impairments, and cognitive decline that can exacerbate the health consequences of maltreatment (Dong, 2015). While older adults have a lower risk of experiencing fraud compared to younger adults, older adults who do experience fraud incur larger financial losses than their younger counterparts (Federal Trade Commission, 2024).

## Barriers to Reporting Maltreatment

In Ontario, Canada, the Long-Term Care Homes Act of 2007 and Retirement Homes Act of 2010 impose a legal duty on any person, including staff, administrators, health professionals and family, to report suspected instances of maltreatment involving residents of retirement or LTC homes. The Fixing Long-Term Care Act of 2021 established additional guidelines for the required care of residents, staffing numbers and training, whistleblower protections and maltreatment reporting, as well as increased accountability and enforcement of regulations.

Many cases of older adult maltreatment do not get referred to the necessary health, social service or legal authorities (Lachs & Berman, 2011). Fraga Dominguez et al. (2021) completed a systematic review of 19 studies that explored help-seeking behavior among older adults who have experienced maltreatment. Several barriers to reporting were identified, including feelings of fear (e.g., retaliation by perpetrator or loss of independence), guilt, shame, stigma and self-blame, as well as a desire to protect the perpetrator. At the same time, older adults with health-related challenges (e.g., Alzheimer's disease) may struggle to acknowledge and communicate the maltreatment. Finally, older adults with limited knowledge of the available support services are less likely to report their experienced maltreatment (Fraga Dominguez et al., 2021).

To date, less is known about why interdisciplinary geriatric care providers fail to report maltreatment they observe in their work settings. Research from Canada, United States and Turkey found that many health professionals have inadequate training and professional practices (e.g., standardized screening measures) that facilitate accurate detection of older adult maltreatment (see Mercier et al., 2020, for a scoping review). Qualitative studies involving health and social work professionals in Iowa (Schmeidel et al., 2012) and Texas (Reingle Gonzalez et al., 2016) found differences among professionals with regards to their self-perceived responsibilities to identify and report older adult maltreatment. Other barriers to reporting included time limitations, a desire to maintain trust and confidentiality, concerns with their training and preparedness, unclear or overly stringent legal definitions of older adult maltreatment, and a lack of knowledge regarding maltreatment reporting procedures (Reingle Gonzalez et al., 2016; Schmeidel et al., 2012). In one of the few Canadian studies (Walsh et al., 2024), older adults and service providers (nurses and social workers) in Alberta highlighted several older adult-specific barriers to reporting maltreatment (e.g., dependency on the perpetrator), but the service providers generally did not discuss their own professional barriers to reporting.

The Department of Justice Canada (2022) completed a review of the older adult maltreatment literature, and conducted interviews with 42 service providers, researchers and government officials. One of the key gaps discovered in the existing literature pertained to the limited Canadian-specific research on older adult maltreatment that takes place in LTC settings. In their systematic review of the literature about older adult maltreatment in health care settings in Canada, Hirst et al. (2016) developed a list of organizational and system-level recommendations for improving the care and well-being of older adults. Some of the primary recommendations included improving staff policies, protocols and training for responding effectively to potential older adult maltreatment.

## Current Study

To better understand why many maltreatment cases do not get reported in community, health or LTC settings, interdisciplinary geriatric care providers from the Greater Toronto area completed a semi-structured qualitative interview that explored their experiences observing situations involving older adult maltreatment, along with their perceptions regarding the common risk factors and perpetrators of maltreatment. These providers also provided information regarding their experiences reporting older adult maltreatment, the barriers to reporting, along with their training and societal recommendations. The qualitative interview approach enabled opportunities for rapport building prior to discussions about this difficult subject matter (Qu & Dumay, 2011). Further, the providers were able to describe their experiences and perspectives in their own words, and the interviewer asked follow-up questions to encourage them to elaborate on their thoughts and ideas. Lastly, these providers completed quantitative questionnaires that objectively assessed their (1) knowledge of the risk factors for older adult maltreatment, (2) attitudes towards intervening in situations involving older adult maltreatment, and (3) self-reported willingness to assess, intervene and report cases of older adult maltreatment. There were no hypotheses regarding the expected findings given the exploratory nature of this predominantly qualitative and descriptive study.

## Methodology

### Participants

Thirty-seven healthcare, social service, LTC, and community interdisciplinary geriatric care providers (*Mage* = 36.8 years, Range: 22 to 60 years old; *n*female = 27; *n*male = 10) from the Greater Toronto Area completed a qualitative interview and four questionnaires for this study. Participants were recruited from local hospitals, LTC and nursing associations in Ontario, as well as through social media advertisements. The current study received ethical approval from the principal investigator's (PI) affiliated university, and all participants provided written and verbal consent. This study also received ethical approval from a local hospital, which then helped recruit health professionals for this study.

Participants included nurses (*n* = 15), social workers (*n* = 4), PSWs (*n* = 4), physicians (*n* = 3), behavior therapists (*n* = 2), and other community professionals (*n* = 9; e.g., dieticians, clinical practice leads, community health workers, and recreation and outreach coordinators) who self-reported that they work with older adults in their communities. The participants worked in hospital and community medical facilities (*n* = 20), nursing homes (*n* = 5), social service agencies (*n* = 4), retirement homes (*n* = 3), and other work settings that provide services to older adults (*n* = 5; e.g., community centers, and home and community care organizations). Most participants indicated that English was their primary language (*n* = 34), and all participants demonstrated the necessary proficiency to communicate in English. Participants identified as Black (29.7%), White (24.3%), Asian (8.1%) or a racial group not specified (21.6%); racial groups that were stated by one participant each included Indigenous, Iranian, Indian and Middle Eastern. Regarding their educational backgrounds, participants either completed a college diploma (*n* = 10), bachelor's degree (*n* = 17), graduate certificate (*n* = 1), master's degree (*n* = 7), or a Doctor of Medicine (MD) degree (*n* = 2). Participants rated their perceptions of their level of expertise in their profession, ranging from 1 (no experience) to 10 (expert in profession). The average self-reported level of expertise was 7.7 out of 10 (*SD* = 1.4; Range = 5 to 10 out of 10).

### Procedure

After signing the consent form, participants completed the qualitative interview virtually using the HIPAA (Health Insurance Portability and Accountability Act) compliant telemedicine platform Doxy.me. Next, participants completed four questionnaires. In addition to a demographics form, participants completed three measures developed by Alipour et al. (2019), including the Potential Risk Factors of Elder Abuse by Family Caregivers Questionnaire, the Attitudes Toward Elder Abuse Phenomenon Questionnaire, and the Performance Self-Assessment Checklist. These questionnaires were either completed during the virtual session or during the participants’ own time. After completing the qualitative interview and four questionnaires, participants were debriefed and received a $20 e-gift card as compensation.

### Materials

#### Qualitative Interview

Participants completed a semi-structured qualitative interview over the telemedicine platform, Doxy.me. All participants were asked the same set of questions; however, follow-up prompts (e.g., “Tell me more about X”) and questions (e.g., “Can you give an example of X?”) were asked to encourage participants to elaborate on their thoughts and ideas. In their review of qualitative interview research, Qu and Dumay (2011) considered the semi-structured interview approach to be the most effective and convenient means of gathering qualitative information. As shown in Table 1, the interview included 14 open-ended questions that were organized into four overarching topics. These topics were informed by prior research findings regarding health professionals’ experiences observing and reporting instances of older adult maltreatment (e.g., Rosen et al., 2017; Yon et al., 2019). The PI—a registered clinical and school psychologist in Ontario who has substantial experience conducting interviews in clinical and research settings— conducted all the qualitative interviews with participants. The median duration of the 37 interviews was 32.1 min, with a range of 16.3 min to 71.0 min.

**Table 1.** Qualitative Interview Topics and Questions.

Topic	Questions
Observations of Older Adult Maltreatment	Please tell me about your occupation (title) and professional responsibilities with older adults? How often have you received reports from/about older adults regarding different forms of maltreatment? What might make you suspicious of older adult maltreatment? What are the common risk factors for older adults who you have witnessed or suspected of being maltreated? Who are the most common perpetrators of older adult maltreatment?
Maltreatment Reporting	What would you do if you became suspicious of older adult maltreatment? What organization(s) do you report any witnessed older adult maltreatment to? What do you think are the most common barriers to reporting maltreatment involving older adults? What are the challenges associated with identifying, talking or asking about maltreatment with older adults?
Professional Training	What training have you received for managing various forms of maltreatment involving older adults, including conversations about legal and/or workplace-based investigating and reporting policies? Are you comfortable with the amount of training you have received so far? What knowledge or training would you like to receive in the area of identifying and reporting older adult maltreatment?
Suggestions for Improving Older Adult Safety	What changes can be made that will help improve the effectiveness of efforts to address older adult maltreatment? Is there anything else you would like to say about improving your ability to address older adult maltreatment?

The Otter.ai transcription tool was used to audio record and transcribe the qualitative interviews. The interviewer also made field notes during the interview and reviewed the accuracy of Otter.ai-generated transcriptions after each interview was completed. Braun and Clarke's (2006) inductive thematic analysis was used to identify the common themes in the participants’ responses. Inductive thematic analysis is a reflexive, bottom-up and exploratory approach wherein there were no pre-existing themes or categories of responses prior to coding the qualitive interviews. Rather, the themes that were generated by the coders were strongly grounded in the participants’ narratives (Braun & Clarke, 2006). In Phase 1 of the coding (familiarization with the data), the PI provided the coders with structured training on inductive thematic analysis, including learning about the coding process, reviewing the PIs coding from another study, and becoming familiarized with the transcriptions from the current study. In Phase 2 (generating initial codes) and 3 (searching for themes), the PI and the two coders read through five participant interviews and identified as many codes as possible. The PI and the coders then met to compare their coding decisions, discuss interpretations of ambiguous or complex participant responses, and combine and refine overlapping themes. This process was repeated for the next five participant interviews. After meeting and combining the re-occurring themes, the two coders then independently coded the remaining 27 interviews and they added new themes that were discussed by participants. For Phase 4 (reviewing themes) and 5 (defining and naming the themes), the coders and PI met to finalize the specifics of each theme and merge any remaining co-occurring themes (Braun & Clarke, 2006).

Cohen's Kappa (κ) and percent agreements were calculated to determine the overall level of agreement between the two coders’ initial scoring of the 37 participant interview transcriptions. Overall, there was a mean Cohen's Kappa (κ) of 0.82 (range = 0.53 to 0.98) for the different interview topics, which indicates “almost perfect” agreement between the two coders (Cohen, 1960). The mean percentage agreement between the two coders was 89.81% (range = 76.50% to 98.92%) for the different interview topics. The two coders met to identify the differences in their coding of the participant interview transcriptions. Next, they recoded the items where they differed in their initial coding and then met again to compare their updated coding of the transcriptions. A “perfect agreement” was achieved (κ = 1.00) between the two coders as they had 100% agreement in their updated coding of the 37 transcriptions. This final dataset was used when discussing the results from the qualitative interviews.

#### Quantitative Measures

On the Demographics Form, participants gave information about their age, gender identity, racial group, level of education, preferred speaking language, and the city that they resided in. Participants also provided information about their occupation, type of facility they worked in, and their self-reported level of experience in their profession.

The Potential Risk Factors of Elder Abuse by Family Caregivers Questionnaire (Alipour et al., 2019) is a 67-item Likert-scale that assessed participants’ abilities to accurately identify different types of older adult maltreatment caused by family caregivers, including physical abuse, sexual abuse, psychological abuse, neglect, and financial exploitation. For each item, participants indicated their level of agreement from 1 (Strongly Disagree) to 5 (Strongly Agree) that each statement was an example of older adult maltreatment. As all 67-items included examples of older adult maltreatment, participants received a score between 1 and 5 depending on their level of agreement for each item. The maximum total score on this measure was 335 (i.e., 67 items X a maximum score of 5 for each item). Alipour et al. (2019) classified scores on this measure into four categories: (1) Excellent Abuse Recognition (total score between 252 and 335), (2) Good Abuse Recognition (total score between 168 and 251), (3) Medium Abuse Recognition (total score between 84 and 167), and (4) Weak Abuse Recognition (total score between 1 and 83). Lastly, Alipour et al., (2019) found significant test-retest stability and ‘good’ internal consistency (α = 0.85, ICC = 0.61) in their psychometric evaluation of this questionnaire.

The Attitudes Toward Elder Abuse Phenomenon Questionnaire (Alipour et al., 2019) was used to measure participants’ attitudes towards intervening in cases involving potential older adult maltreatment. This questionnaire consisted of six Likert-scale items whereby participants were asked to indicate their level of agreement with statements that explored their attitudes towards asking questions and intervening in cases of suspected older adult maltreatment. Three items were scored based on how much they agreed with each statement (e.g., “It is my duty to intervene in elder abuse cases”), from a score of 1 (Strongly Disagree) to 5 (Strongly Agree). Conversely, three items were reversed scored according to how much they disagreed with the statement (e.g., “It is useless to report an elder abuse case when the elderly victim is denying it”). The maximum score on this measure was 30 with higher scores indicating a stronger performance. Alipour et al. (2019) reported that this measure had face and content validity, along with ‘high’ test-rest reliability (α = 0.83).

The Performance Self-Assessment Checklist (Alipour et al., 2019) included 13 Likert-scale items that assessed participants’ self-reported willingness to assess, intervene and report instances of older adult maltreatment. Participants indicated their level of agreement, from Strongly Disagree (score of 1) to Strongly Agree (score of 5), to 13 statements that explored how they respond to instances of observed older adult maltreatment. The maximum score on this measure was 65, and higher scores reflecting a higher degree of self-reported work assessing, intervening and reporting instances of observed older adult maltreatment. Alipour et al. (2019) reported that this measure had face and content validity; however, the test-rest reliability score of 0.65 fell in the ‘questionable’ reliability range.

## Results

The results section is organized according to the four overarching topics discussed during the qualitative interviews (see Table 1). For each topic, the frequency that each subtheme was communicated by participants will be presented in the text and relevant tables. Verbatim participant quotes will be provided as illustrative examples for some subthemes, and square brackets will be used to indicate instances where filler words have been added or removed.

## Observations of Older Adult Maltreatment

Participants discussed their professional responsibilities, and their experiences observing older adult maltreatment in their workplace. Although most participants (*n* = 35; 94.6%) worked directly with older adults, few participants reported having experience in the areas of assessment (*n* = 5; 13.5%), reporting (*n* = 5; 13.5%), and interventions (*n* = 3; 8.1%) for situations involving older adult maltreatment. Furthermore, 24 out 37 participants (65%) reported observing a form of older adult maltreatment on a “frequent” basis, including on a daily (*n* = 4; 10.8%), weekly (*n* = 10; 27.0%) or monthly (*n* = 6; 16.2%) basis. For example, P23 stated that they observe older adult maltreatment “very often, probably an average of one or two in a day. On some days, we have [a] very high [number of] cases, probably five and above.”

### Indicators

Participants explained what might make them suspicious that an older adult was being maltreated. Twenty participants (54.1%) discussed indicators of older adult physical abuse, such as forceful confinement, physical hitting, as well as odd wounds, bruises (e.g., pressure sores) and fractures. Seventeen participants (46.0%) highlighted indicators of neglect, including a caregiver having limited knowledge and training about caring for older adults with special needs (e.g., leaving an older adult at home unattended all day); older adults with notable hygiene and nutrition issues, unexpected weight loss, and frequent falls; staff with overly high caseloads, and thus, they have “taken shortcuts” when caring for each person; and a caregiver failing to provide an older adult with their medications. Eleven participants (29.7%) provided examples of psychological abuse, including witnessing other staff and/or caregivers speak aggressively and disrespectfully towards people in their care; family caregivers isolating the older adult from other family members and service providers; power imbalances between an older adult and their caregiver; and the older adult being fearful of going home.

Regarding indicators of financial abuse, ten participants (27.0%) noted that some caregivers took on the role of a Power of Attorney (POA) even though the older adult was fully capable of making financial decisions. Other participants reported observing odd spending habits by the older adult, as well as caregivers misusing their funds. Three participants (8.1%) highlighted indicators of sexual abuse, including uncharacteristic urinary tract infections and sexually transmitted infections, and finding another resident lying in bed with a fearful older adult. Finally, three participants (8.1%) stated that older adults who showed uncharacteristic aggression towards staff and/or signs of trauma may have been maltreated recently.

### Risk Factors

Participants were asked to discuss the risk factors for older adult maltreatment. Approximately two-thirds of participants (*n* = 25; 67.6%) indicated that older adults who were dependent on their caregivers because of cognitive, physical or financial challenges were at risk for maltreatment. With regards to older adults with cognitive-related challenges (*n* = 20; 54.1% of responses), P4 explained that these older adults may have a heightened risk for maltreatment because “they may not be able to recognize or communicate when they are being maltreated.” Regarding family-related risk factors (*n* = 17; 46% of responses), P18 stated that “missed appointments [and] resistance from families to involve community support [is] a big red flag.” Other cited family-related risk factors included older adults with family members who had addiction and other mental health challenges, as well as families with limited finances to manage the older adults’ care. Insufficient caregiving training, education, resources and time (e.g., overly high caseloads) was another frequently endorsed risk factor (*n* = 15; 40.5% of responses). Lastly, older adults who appear lonely, withdrawn and isolated (*n* = 10; 27% of responses), as well as those with mobility challenges (*n* = 8; 21.6% of responses), were also cited as being at-risk for maltreatment, according to participants. Refer to Table S1 in the Supplementary Materials for an overview of the risk factors for older adult maltreatment discussed by participants.

### Perpetrators

Participants next discussed the common perpetrators of older adult maltreatment. Most participants (*n* = 26; 70.3%) identified a family member as a common perpetrator of older adult maltreatment. This was particularly evident when the adult child (*n* = 9; 24.3%), spouse/domestic partner (*n* = 8; 21.6%), grandchild (*n* = 4; 10.8%) or sibling (*n* = 3; 8.1%) was the primary caregiver. Participant 1 stated that “family, children and grandchildren [are the perpetrators in] 90% of the cases. Which is why, you know, seniors are often so reluctant to report or complain.” Whereas P12 explained that maltreatment perpetrators are often the “caregivers, grandchildren and older children, especially when they don’t take care of them well. They go to work and leave the older adult alone, who has difficulty going to the bathroom by themselves.” Nearly half the participants (*n* = 17; 46.0%) reported that they observed other interdisciplinary geriatric care providers perpetrate older adult maltreatment, including PSWs, nurses, LTC management, optometrists, and unregulated care staff. Participant 8 stated that “PSWs bring their home problems, like a breakup or children driving them up the wall, into work and won’t offer good service to our residents.” On the other hand, P3 indicated that “we can’t blame them [PSWs] fully because of shortages of staff. [For example,] two workers for 30 seniors. Dealing with 5 resident calls at a time. Sometimes they [the PSWs] are helpless too.” Some participants indicated that friends, neighbors and other LTC residents (*n* = 10; 27.0%), as well as strangers or unfamiliar persons (*n* = 5; 13.5%), are common perpetrators.

### Maltreatment Reporting

Participants explained what they would do if they became suspicious of older adult maltreatment, including the types of organizations they would reach out to. Most participants (*n* = 36; 97.3%) indicated that they would report observed older adult maltreatment, and over half (*n* = 21;56.8%) stated that they would directly assist the older adult. Twenty-one participants (56.8%) indicated that they would report the observed maltreatment to their supervisor, manager, director of care, and/or lead (or charge) nurse. Approximately half the participants indicated that they would report the maltreatment to external (*n* = 19; 51.4%) or internal (*n* = 18; 48.7%) support services, and/or to emergency services (*n* = 18, 48.7%; e.g., police, health and fire emergency services). Several external support services were identified, including mandatory reporting locations (e.g., ministries of health and LTC), professional colleges when the abuser is a health professional, and consultation with external social workers, medical professionals, and Elder Abuse Prevention Ontario. Among internal support services, participants reported consulting with elder abuse team members, social workers, psychiatrists and physicians who worked at their organization. Lastly, nine participants (24.3%) indicated that they would discuss their concerns with the non-offending family members, caregivers or substitute decision-makers. Participant 18 explained that:

“When it comes to involvement with someone who's incapable, it really does involve many community partners to address [the maltreatment]. So, it may involve the police, it may involve law [or] fire [services], [and] it may involve Public Guardian and Trustee. It may involve a medical practitioner to determine capacity. So, the approach is quite different. Maybe we're looking to a substitute decision-maker or something like that to determine what we do next.”

#### Barriers to Maltreatment Reporting

Participants next described the barriers to reporting maltreatment, including factors that might discourage older adults, themselves and other interdisciplinary geriatric care providers from reporting. As shown Table 2, fear of the consequences of reporting the maltreatment, both for the older adult and the geriatric care provider, was reported by most (*n* = 31; 83.8%) participants. For example, P30 stated that they’ve “had some clients say that they don’t want to lose housing if the worker, who is the abuser, has an office there. [They] don’t want to be cut off from food and [other] resources.” Other participants indicated that they may be fearful of losing friendships with their coworkers, being perceived as a whistleblower, and/or be penalized by their management for reporting. Participant 32 shared that some providers may be “scared they will lose their jobs if they report or be seen as un-hireable [or] as a sellout. Your job shouldn’t be at-risk for saving a person's life.”

**Table 2.** Thematic Analysis: Barriers to Reporting Older Adult Maltreatment.

Barrier to Reporting	Number of Participants (%)	Response Examples
Fear of Consequences	*n* = 31 (83.8%)	Older adult fear of losing independence and autonomy. Professional fear of being perceived as a whistleblower or being punished.
Older Adult Apprehension	*n* = 17 (46.0%)	Feelings of shame, guilt, embarrassment, helplessness and denial. Desire privacy or do not want to bother others. Perceive reporting as unhelpful.
Limited Rapport	*n* = 14 (37.8%)	Older adult and caregiver do not trust the geriatric care provider.
Insufficient Knowledge	*n* = 13 (35.1%)	Being unsure about whether to report maltreatment. Uncertainty about specific organizations to report to.
Desire to Protect Perpetrator	*n* = 11 (29.7%)	Fear of perpetrator being incriminated. Defending or minimizing perpetrator's abusive behaviors.
Professional Barriers	*n* = 11 (29.7%)	Insufficient staff training and experience on identifying and reporting maltreatment. Limited training about cultural responsivity and microaggressions. Emotional impact of asking about maltreatment.
Institutional Barriers	*n* = 10 (27.0%)	Supervisor not following up with initial report. Insufficient time to report. Large caseloads. Other staff are unapproachable or unwilling to help.
Societal Barriers	*n* = 9 (24.3%)	Ageism; public permissiveness of older adult maltreatment.
Victim Cognitive Challenges	*n* = 9 (24.3%)	Communication, memory and decision-making challenges.

Nearly half the participants (*n* = 17; 46.0%) reported that older adult apprehension, such as feelings of shame, guilt, embarrassment and helplessness, can discourage them from reporting. Similarly, some participants shared that older adults may be apprehensive to report maltreatment because they want to keep it a private matter, as well as if they believe that reporting it will be unhelpful. Fourteen participants (37.8%) indicated that it is difficult to discuss the topic of older adult maltreatment when they have limited trust and rapport with the older adult and/or caregiver. Additionally, older adults may not report their experienced maltreatment due to their desire to protect and/or defend the perpetrator (*n* = 11; 29.7% of responses), such as protecting their adult child, grandchild, domestic partner or spouse. Some participants shared that cognitive-related challenges (*n* = 9; 24.3% of responses) in the areas of communication (e.g., not being able to verbalize the maltreatment), memory (forgetting that the maltreatment occurred), and decision-making (e.g., not being able to identify that they are being maltreated) can limit an older adult's abilities to tell others about the abuse and/or neglect. Further, nine participants (24.3%) argued that ageist perspectives and permissiveness towards the topic of older adult maltreatment in society discourages reporting.

Some professional and institutional barriers to reporting were identified. Thirteen participants (35.1%) reported that they felt they had insufficient knowledge about when and/or where to report the maltreatment. Further, limited cultural responsivity training can make it difficult to identify microaggressions, and discussing the topic of older adult maltreatment can negatively impact the mental health of interdisciplinary geriatric care providers. Participant 22 shared that reporting maltreatment is:

“Unknown for me. I don't know if I call [and] reach out, would they then collect my information? What consequence would be for me [if] I didn't? But honestly, how many times I wanted to do it [make a report], [yet] I had some people that said they ‘actually reported [maltreatment] but nothing happened.’”

Among the professional barriers, eleven participants (29.7% of responses) noted older adult maltreatment may not get reported due to staff inexperience and/or insufficient training. Participant 10 stated that some providers:

“Don't know any different because their training isn't standardized. They would just continue it [the abusive behaviour], especially when it's a task-oriented thing and you got to get people done. Pushing, shoving, being very rough with individuals in their hygiene. They just thought it was doing a task, and until someone witnesses [the maltreatment] outside of their group, [they] wouldn't know.”

Lastly, institutional barriers (*n* = 10; 27% of responses) included having overly large caseloads to devote the necessary time to report observed maltreatment, along with low confidence that their supervisors and coworkers would assist them.

### Professional Training

Participants described their training background pertaining to managing situations involving older adult maltreatment. Two-thirds (*n* = 25; 67.6%) of participants indicated that they received training about older adult maltreatment at their workplace, such as during their orientation training, refresher trainings, workshops and seminars. Thirteen participants (35.1%) pursued education about older adult maltreatment outside their workplace, including attending professional conferences, courses, seminars and workshops. Seven participants (18.9%) reported receiving training about older adult maltreatment during their college, university or post-graduate education. Finally, two participants (5.4%) received training through mentorship and/or supervision.

Participants were asked if they were comfortable with the amount of training they received so far for managing situations involving older adult maltreatment. Overall, 23 participants (62.2%) indicated that they were comfortable with the amount of training they had received, 13 (35.1%) stated they were not comfortable with their training, and 1 participant (2.7%) was unsure. Participant 2 stated that “yes, I’m comfortable with [the] amount of training. [However], I wish there was more ongoing training because I know that the way that a person presents signs of abuse may change.” Conversely, P21 was not comfortable with the training they had received because they could “barely remember last time I got education in the area.”

#### Training Recommendations

Participants discussed the training they would like to receive in the area of identifying and reporting older adult maltreatment. As seen in Table S2 in the Supplementary Materials, nearly half (*n* = 18; 48.7%) the participants wanted more training about how to effectively identify, assess, respond-to and report observed older adult maltreatment. Thirteen participants (35.1%) shared that they wanted more frequent refresher trainings, along with workplace-specific trainings (e.g., responding to maltreatment in hospital settings). Ten participants (27%) wanted more knowledge about community referral resources for at-risk older adults, including legal, social and health support services. Seven participants (18.9%) shared that they wanted best practice interviewing training, including rapport-building strategies for establishing trust and interviewing practices that encourage open discussions about suspected maltreatment. For instance, P17 wanted to learn about:

“Having courageous conversations with families and being better at assessing for the maltreatment, in terms of physical [and] sexual abuse. I would find that very difficult to talk about. I've tried to talk about it. Almost you need someone to like watch what you're doing to give you feedback.”

Furthermore, some participants (*n* = 4; 10.8%) expressed that they wanted more training about legal rights pertaining to older adult decision-making capacity and improved caregiving training (*n* = 2; 5.4%).

#### Knowledge of Risk Factors for Older Adult Maltreatment

Descriptive analyses were used to analyze participant performance on the Potential Risk Factors of Elder Abuse by Family Caregivers Questionnaire (Alipour et al., 2019). The median total score for the sample was 259 out of 335, with a range of 87 to 334 out of 335. Most scores on this measure either fell into the ‘Excellent’ (64.9%) or ‘Good’ (29.7%) recognition of elder abuse symptoms ranges, and two participants (5.4%) scored in the ‘Medium’ recognition range. Bivariate correlation analyses revealed no significant correlations between years of experience, self-reported level of experience, and total scores on this quantitative measure.

#### Attitudes and Willingness to Intervene in Cases of Older Adult Maltreatment

Descriptive analyses examined participant performance on the Attitudes Toward Elder Abuse Phenomenon Questionnaire (Alipour et al., 2019). Participant median score on this measure was 24 out of 30, with a range of 15 to 30 out of 30. On the Performance Self-Assessment Checklist (Alipour et al., 2019), participants’ median score on this measure was 52 out of 65, with a range of 41 to 64 out of 65. Bivariate correlation analyses revealed no significant correlations between years of experience, self-reported experience level, and total scores on these two quantitative measures.

### Suggestions for Improving Older Adult Safety

To conclude the interview, participants gave suggestions for improving older adult safety. Six themes related to professional, government policy, and societal recommendations emerged from the inductive thematic coding of the participant interviews. Two-thirds of participants (*n* = 25; 67.6%) shared that they wanted increased public awareness about the growing problem of older adult maltreatment, risk factors for maltreatment, reporting guidelines, and older adult caregiving training for family members. Some participants highlighted that there is ongoing societal discrimination towards older adults, and that further public education is needed about respecting the dignity of this population.

Twenty participants (54.1%) advocated for government policy changes, particularly with respect to increased funding for OA-specific programs, interdisciplinary geriatric care providers (i.e., higher income and more staff), and older adults who require financial support. Some participants argued that there needs to be improved government regulations and oversight for formal and family caregivers, and power of attorneys. Further, some argued for improved collaboration between government ministries (e.g., Ministry of Health) and geriatric care providers. Nineteen participants (51.4%) reiterated the importance of enhancing geriatric care provider training in the areas of identifying and reporting older adult maltreatment, and attitudes towards older adult caregiving.

Nine participants (24.3%) advocated for improved older adult maltreatment reporting guidelines. For instance, they recommended more streamlined and standardized reporting procedures (e.g., one specific online and/ phone reporting location) for all interdisciplinary geriatric care providers. Participant 7 suggested that there should be “an objective third party that staff can make reports to about abuse or that residents can report their abuse to. [The objective third party] would have no interest in the operations of the facility.” Seven participants (18.9%) reiterated the importance of developing standardized guidelines for helping interdisciplinary geriatric care providers to effectively interview older adults at-risk for maltreatment. Lastly, seven participants (18.9%) highlighted the need for reduced caseloads for interdisciplinary geriatric care providers to increase the amount of time they can spend with each resident, provide them with breaks, and improve their overall quality of care. Refer to Table S3 in the Supplementary Materials for an overview of the recommendations for improving older adult safety and well-being.

## Discussion

Older adult maltreatment frequently occurs in health and LTC settings (see Yon et al., 2019), and many of these cases do not get reported to the necessary health, social service or legal authorities (Lachs & Berman, 2011). While much is known about the older adult-specific barriers to reporting (see Fraga Dominguez et al., 2021, for a systematic review), less is known about why interdisciplinary geriatric care providers may be unwilling or unable to report the abuse and neglect they observe. Systematic reviews over the last decade revealed that there is limited Canadian research on older adult maltreatment in LTC settings (Department of Justice Canada, 2022), and there is a need for improved staff policies, protocols and training (Hirst et al., 2016). To improve geriatric care provider reporting protocols and training, it is necessary to explore their experiences, knowledge and reporting practices in situations involving older adult maltreatment. Therefore, the objectives of the current mixed-methods study were to explore Ontario interdisciplinary geriatric care providers’ 1) experiences observing and reporting older adult maltreatment, 2) barriers to reporting, 3) training experiences and needs, and 4) recommendations for improving older adult safety.

### Observations of Older Adult Maltreatment

Consistent with the meta-analytic findings from Yon et al. (2019), nearly two-thirds of interdisciplinary geriatric care providers in the current study reported observing older adult maltreatment on a ‘frequent’ basis, with 39% observing these instances on at least a weekly basis. Nevertheless, the current findings pertain to non-documented observations of older adult maltreatment. Given that there were 781 total cases of older adult maltreatment discovered during inspections of 627 LTC homes in Ontario from 2019 to 2022 (Baumann et al., 2024), it appears that the majority of staff observations of older adult maltreatment in these settings do not get discovered or recorded during the mandatory LTC home inspections by the Ontario Ministry of Long-Term Care.

Consistent with prior research (see Jackson, 2016, for a review), many interdisciplinary geriatric care providers shared that family members and other health professionals are common perpetrators of older adult maltreatment. This underscores why many older adults are reluctant to disclose the maltreatment given that the perpetrator is often someone they have a close and familial bond with and/or it is a person of authority who is responsible for their care. Older adult dependency on their caregivers was noted by most participants as a risk factor for maltreatment, particularly in cases in which the older adult has cognitive, mobility, and/or other health challenges. If a caregiver is unprepared for the difficult responsibility of caring for an older adult with complex needs, including long-hours and stress associated with caregiving due to having insufficient training, limited time, financial resources and/or support (Arriagada, 2020), the risk for unintentional neglect to older adults in their care increases. This is reflected in the findings from Akhtar-Danesh et al. (2022), who found that 27% of 106,765 LTC residents in Ontario demonstrated at least one indicator of neglect (e.g., dehydration and urinary tract infections).

The findings from the current study and other Canadian research (Wyman et al., 2025) indicate that family members are the most common perpetrators of older adult maltreatment, in part due to their lack of training, time, and general preparedness to be caregivers. Meanwhile, nearly half of the interdisciplinary geriatric care providers highlighted that a history of family problems increases the risk for older adult maltreatment. Given that many Canadians are becoming family caregivers for their aging family members (Statistics Canada, 2018), there is a tremendous need for low-cost and accessible caregiver education programs, home-based care and respite services, and counseling services to support these caregivers (Hirst et al., 2016).

### Maltreatment Reporting Barriers

Five themes of older adult maltreatment reporting practices were highlighted by the interdisciplinary geriatric care providers. Approximately half the providers stated that they would report their concerns to a supervisor, contact an external support service (e.g., Ministry of Long-Term Care), consult with a support professional (e.g., social worker) at their organization, and/or contact emergency services. While notifying supervisors or directors is recommended for health professionals (e.g., Registered Nurses Association of Ontario, 2014) and LTC staff (e.g., Fixing Long-term Care Act, 2021), nearly half the providers in the present study did not indicate they would contact the appropriate government agencies or emergency services when they suspected maltreatment. Reporting observed maltreatment in LTC and retirement home settings is legally mandated in Ontario (Fixing Long-Term Care Act, 2021). Further, delaying the reporting of older adult maltreatment can result in ongoing and serious harm, particularly for older adults with pre-existing health conditions and complex needs (Burnett et al., 2016; Dong et al., 2009).

Consistent with prior older adult research (Fraga Dominguez et al., 2021), the providers shared that older adults can be apprehensive to report their experienced maltreatment due to feelings of fear, shame, guilt, embarrassment, helplessness and denial, as well as a desire to protect the perpetrator (see Table 2). Further, some providers shared that older adults may have distrust towards the societal support systems, as well as cognitive-related challenges that make acknowledging and reporting the maltreatment difficult. Many of these older adult-specific barriers to reporting are also evident in other age demographics, including child victims of sexual abuse (Lemaigre et al., 2017) and younger adults who have experienced domestic violence (Heron & Eisma, 2021).

The interdisciplinary geriatric care providers shared their own apprehensions towards reporting older adult maltreatment. Several providers expressed being fearful of being punished by their superiors and/or being seen as a whistleblower. In addition, some providers expressed low confidence in their preparedness to effectively identify and report the maltreatment, as well as concerns with having insufficient time and institutional support. As some of these reporting barriers were also endorsed by staff in Iowa (Schmeidel et al., 2012) and Texas (Reingle Gonzalez et al., 2016), it is clear that systemic changes are needed to overcome these obstacles to older adult maltreatment reporting. Access to evidence-based geriatric training has shown to result in increased maltreatment identification and reporting among interdisciplinary geriatric care providers (see Ranabhat et al., 2022, for a systematic review). Furthermore, it is recommended that interdisciplinary geriatric care providers have access to third-party (or outside organization) social workers to consult with, organizational adherence to the whistleblower protections present in the Fixing Long-Term Care Act of 2021, and the necessary time to adequately support at-risk older adults.

### Improving Older Adult Safety: Training Needs and Societal Recommendations

There was a wide range of self-reported training experiences and education about older adult maltreatment, such that many received workplace-specific training, and some received training on their own time, during their post-secondary education, and/or through mentorship. When asked if they were comfortable with their amount of training to date, just over 60% of providers indicated that they were comfortable and over one-third stated they were not comfortable. When assessing their knowledge of the risk factors for older adult maltreatment, most providers scored within the ‘Excellent’ or ‘Good’ abuse recognition ranges with the total median score (259 out of 335) corresponding with the mean score of Canadian law enforcement professionals (*M* = 265; Wyman et al., 2025) and Iranian nurses (*M* = 256; Ghaffari et al., 2020) on the same measure. Moreover, the providers’ median scores on the Attitudes Toward Elder Abuse Phenomenon Questionnaire and Performance Self-Assessment Checklist indicates that most of these providers believe that it is their duty to intervene in cases of older adult maltreatment.

Participants shared several professional training and societal recommendations for increasing the safety of older adults, including improved training about how to effectively identify, assess, and report instances of older adult maltreatment. To improve older adult safety, many indicated that the general public needs more education about older adult caregiving, maltreatment indicators and reporting processes, as well as the detrimental impacts of ageism. Further, several professionals advocated for government policy changes (e.g., increased funding for geriatric health and LTC services), as well as improved caregiver training requirements.

The quantitative results suggest that most providers in this study have appropriate knowledge of the risk factors and indicators of older adult maltreatment, as well as a sense of personal responsibility to intervene to support at-risk older adults. Conversely, the qualitative findings suggest there is notable variation among these providers in their self-confidence, training needs, and perceptions of professional and institutional support systems for managing situations involving potential older adult maltreatment. While there are training and education resources available in Ontario, such as through Elder Abuse Prevention Ontario, it is clear that these programs should become more standardized and easily accessible to interdisciplinary geriatric care providers.

Importantly, there is a great need for increased government funding in order to train and educate more interdisciplinary geriatric care providers. Canada will need to double their LTC capacity by 2035 (Conference Board of Canada, 2017), and there are currently thousands of unfilled positions in health occupations (Government of Canada, 2024). While the Government of Ontario (2024) proposed that it plans to build 58,000 new or upgraded LTC beds by 2028, there are considerable challenges with hiring and retaining interdisciplinary geriatric care providers. A Government of Ontario study (2020) found that 25% of PSWs with two or more years of experience leave the sector each year in Ontario due to high caseloads, long working hours, inadequate pay, and a lack of support. Without addressing the disparities in geriatric care training, coupled with the high burnout and turnover in these professions, the quality of older adult care will continue to be compromised in these settings.

The present findings suggest that many of the participating geriatric care providers are advocating for a broader professional and organizational cultural change with regards to the process and outcomes of reporting older adult maltreatment. For example, several participants indicated that they were hesitant to report observed maltreatment because of unclear internal reporting procedures, fear of professional or personal repercussions, limited belief that there would be any meaningful outcomes from reporting, as well as feeling rushed and unsupported during the reporting process. Being accurate in identifying indicators of maltreatment is essential; however, it is equally important that these providers feel confident, protected, and institutionally supported when reporting their concerns about an older adult's safety and wellbeing. Refer to Table 3 for a review of the integrated qualitative and quantitative findings in the domains of *older adult maltreatment knowledge*, *willingness to report*, and *professional training needs*.

**Table 3.** Mixed Method Integration of the Qualitative and Quantitative Findings.

Domain	Qualitative Themes	Quantitative Findings	Interpretation
Knowledge	Some professionals reported low self-confidence in their training preparedness to manage situations involving maltreatment. Inconsistency in older adult maltreatment reporting locations and procedures.	“Excellent” to “Good” recognition of maltreatment risk factors on a quantitative questionnaire. Self-reported level of experience did not correlate with maltreatment risk factor identification accuracy.	Objective knowledge of maltreatment risk factors may not translate into perceived confidence or preparedness to manage situations involving observed maltreatment.
Willingness to Report Maltreatment	Many shared that they would either report maltreatment or directly assist at-risk older adults. Several professional and institutional barriers to reporting were identified that can discourage maltreatment reporting.	Scores on two quantitative measures indicate that most of the participating inter-disciplinary geriatric care providers believe that it is their duty to intervene in cases of older adult maltreatment.	Professional sense of duty to report is high; however, institutional and professional barriers can make the process of reporting challenging. Professional and organizational culture change is needed with regards to the process and outcomes of reporting.
Training Needs	Identifying, assessing and reporting maltreatment. Knowledge of community referral services. Refresher training opportunities.	High accuracy identifying risk factors for older adult maltreatment. High sense of personal duty to support at-risk older adults.	Providers are well equipped to identify risk factors for maltreatment. Future training should focus on standardized, efficient, and protected reporting of older adult maltreatment.

### Limitations

The current mixed methods study explored the experiences and perspectives of interdisciplinary geriatric care providers in the Greater Toronto area. The current sample size of 37 exceeds similar qualitative studies involving interdisciplinary geriatric care providers, including Reingle Gonzalez et al. (2016; *N* = 23), Schmeidel et al. (2012; *N* = 23), and Walsh et al. (2024; 10 older adults and 10 service providers). While the current sample included interdisciplinary geriatric care providers with diverse racial identities, more research is needed that explores the perspectives of interdisciplinary geriatric care providers who work in rural and Indigenous communities. This research is necessary given that these communities often have fewer community and health resources for older adults, as well as greater staff shortages (Canadian Institute of Actuaries, 2024). Further, it is important to explore the socio-cultural factors that might influence the reporting of maltreatment, particularly for cultural groups who have lower trust and confidence in community support services (e.g., Indigenous communities; Chrismas, 2012).

### Directions for Future Research

Many interdisciplinary geriatric care providers in the present study indicated that they wanted improved training pertaining to identifying, assessing and reporting older adult maltreatment. In their systematic literature review of 14 studies, Ranabhat et al. (2022) found that nursing students, registered nurses and sexual assault nurse examiners who received mixed method learning opportunities (e.g., face to face lectures, case scenarios and debriefings) developed more knowledge about older adult maltreatment, along with increased maltreatment identification accuracy and willingness to make a report. Nevertheless, Ranabhat et al. (2022) indicated that there currently is limited research regarding the long-term positive outcomes of these trainings for preventing instances of maltreatment in LTC settings. Finally, it essential to gather the perspectives of older adults about the facilitators to reporting maltreatment, particularly in situations wherein the maltreatment is perpetrated by a caregiver

### Conclusions

The present mixed methods study represents one of the few Canadian investigations into interdisciplinary geriatric care providers’ experiences when responding to situations involving older adult maltreatment. Results from the quantitative measures indicate that the participating interdisciplinary geriatric care providers had the appropriate knowledge of maltreatment risk factors and they are motivated to help at-risk older adults. However, integration of the qualitative data reveals a more complex picture. Several older adult-specific and professional barriers to reporting their maltreatment observations were identified during the qualitative interview. For example, fear of consequences (e.g., being perceived as a whistleblower), limited rapport, insufficient training and institutional obstacles (e.g., large caseloads and a lack of management support) can discourage them from reporting observed older adult maltreatment. At the same time, many providers highlighted the need for more access to targeted training programs and systematic supports that will facilitate safe and effective reporting of observed maltreatment. Altogether, meaningful professional and institutional culture change is very much needed—particularly regarding the reporting processes, professional and institutional support systems, training and psychological safety—to empower these interdisciplinary geriatric care providers to intervene confidently and effectively in cases of suspected older adult maltreatment.

## Supplemental Material

sj-docx-1-cjn-10.1177_08445621261443833 - Supplemental material for Responding to Older Adult Maltreatment: Interdisciplinary Geriatric Care Provider Experiences and Training Needs Supplemental material, sj-docx-1-cjn-10.1177_08445621261443833 for Responding to Older Adult Maltreatment: Interdisciplinary Geriatric Care Provider Experiences and Training Needs by Joshua Wyman, Cassandre Dion Larivière, Tala Tayem and Lindsay Malloy in Canadian Journal of Nursing Research
