# Supplementary material for: Responding to Older Adult Maltreatment: Interdisciplinary Geriatric Care Provider Experiences and Training Needs
Source: Can J Nurs Res. 2026 Apr 20;58(3):398–412. doi: 10.1177/08445621261443833 (PMC13454408; doi:10.1177/08445621261443833)
Supplement: Supplementary file 1 [file sj-docx-1-cjn-10.1177_08445621261443833.docx]

**Supplementary Materials**

**Table S1**

*Thematic Analysis: Risk Factors for Older Adult Maltreatment*

| **Risk Factor** | **Number of Participants (%)** | **Response Examples** |
| --- | --- | --- |
| Dependency | *n* = 25 (67.6%) | Older adults who are unable to take care of themselves; require considerable support and care from their caregivers and/or service providers; financial dependency on perpetrators. |
| Cognitive Challenges | *n* = 20 (54.1%) | Dementia; memory and communication challenges; limited decision-making capacity; unable to recognize that they are being maltreated; frequent wandering. |
| Family Problems | *n* = 17 (46.0%) | Family members with addiction and other mental health challenges. Older adults who rather be at the hospital then at home with family. Family members who isolate older adults from service providers; family financial issues. |
| Insufficient Caregiving | *n* = 15 (40.5%) | Limited training and education about older adult caregiving; insufficient time and finances; staff who feel rushed and who have high caseloads. |
| Isolation | *n* = 10 (27.0%) | Older adults who isolate themselves from others; older adults with few friends, family and social supports; older adults who are lonely and withdrawn. |
| Mobility Challenges | *n* = 8 (21.6%) | Older adults who have difficulty leaving home, those confined to their beds or wheelchairs, and those at-risk for falls. |

**Table S2**

*Thematic Analysis: Training Recommendations*

| **Training Recommendations** | **Number of Participants (%)** | **Response Examples** |
| --- | --- | --- |
| Responding to Older Adult Maltreatment | *n* = 18 (48.7%) | Identification, assessment and reporting of older adult maltreatment. |
| Refresher Trainings | *n* = 13 (35.1%) | More frequent trainings about identifying and reporting maltreatment. Workplace-specific trainings. Free webinars and workshops. |
| Referral Resources | *n* = 10 (27.0%) | Knowledge about community support services for at-risk older adults. |
| Interviewing Training | *n* = 7 (18.9%) | Speaking to victims about suspected maltreatment. Rapport-building strategies. |
| Older Adult Legal Rights | *n* = 4 (10.8%) | Legal rights in situations involving suspected maltreatment. Decision-making capacity. |
| Older Adult Caregiving | *n* = 2 (5.4%) | Improved caregiving training for older adults with special needs. |

**Table S3**

*Thematic Analysis: Recommendations for Improving Older Adult Safety*

| **Recommendations** | **Number of Participants (%)** | **Response Examples** |
| --- | --- | --- |
| Public Awareness & Education | *n* = 25 (67.6%) | Improved public knowledge and awareness of indicators of maltreatment, older adult caregiving and safety, ageism, reporting guidelines, and respect for the dignity of this population. |
| Government Policy Changes | *n* = 20 (54.1%) | Increased funding for social and health programs, and older adults who require financial support. Funding for more staff and higher staff compensation. Collaboration between government and service providers. Increased government regulations for caregivers and Power of Attorneys. |
| Improved Staff Training | *n* = 19 (51.4%) | Training about identifying and reporting older adult maltreatment. Improving staff attitudes towards caregiving. More consistent and ongoing training. |
| Maltreatment Reporting Guidelines | *n* = 9 (24.3%) | Consistent reporting guidelines. Objective third parties to receive reports of maltreatment. Standardized reporting portals. |
| Interviewing Guidelines | *n* = 7 (18.9%) | Interviewing question guidelines for asking older adults about maltreatment. |
| Reduced Caseloads | *n* = 7 (18.9%) | Smaller caseloads and workload for professionals. More opportunities to take breaks. |
